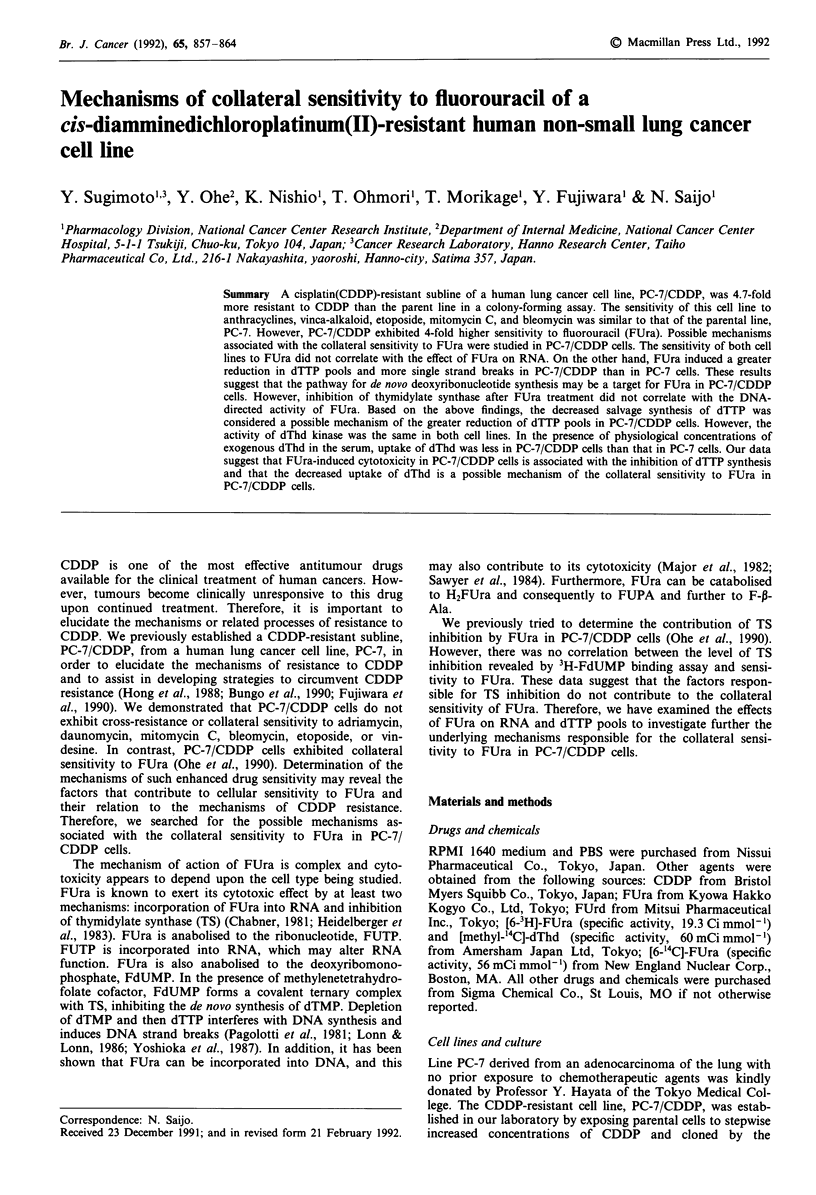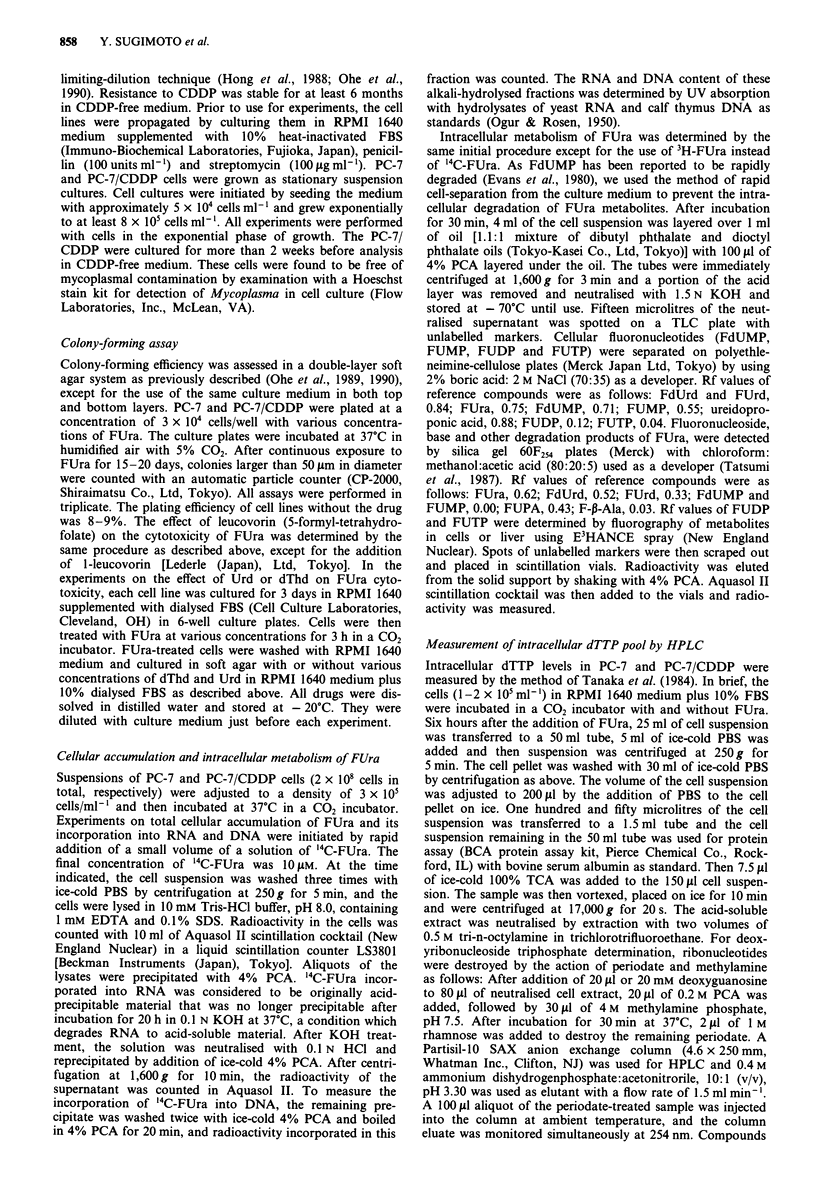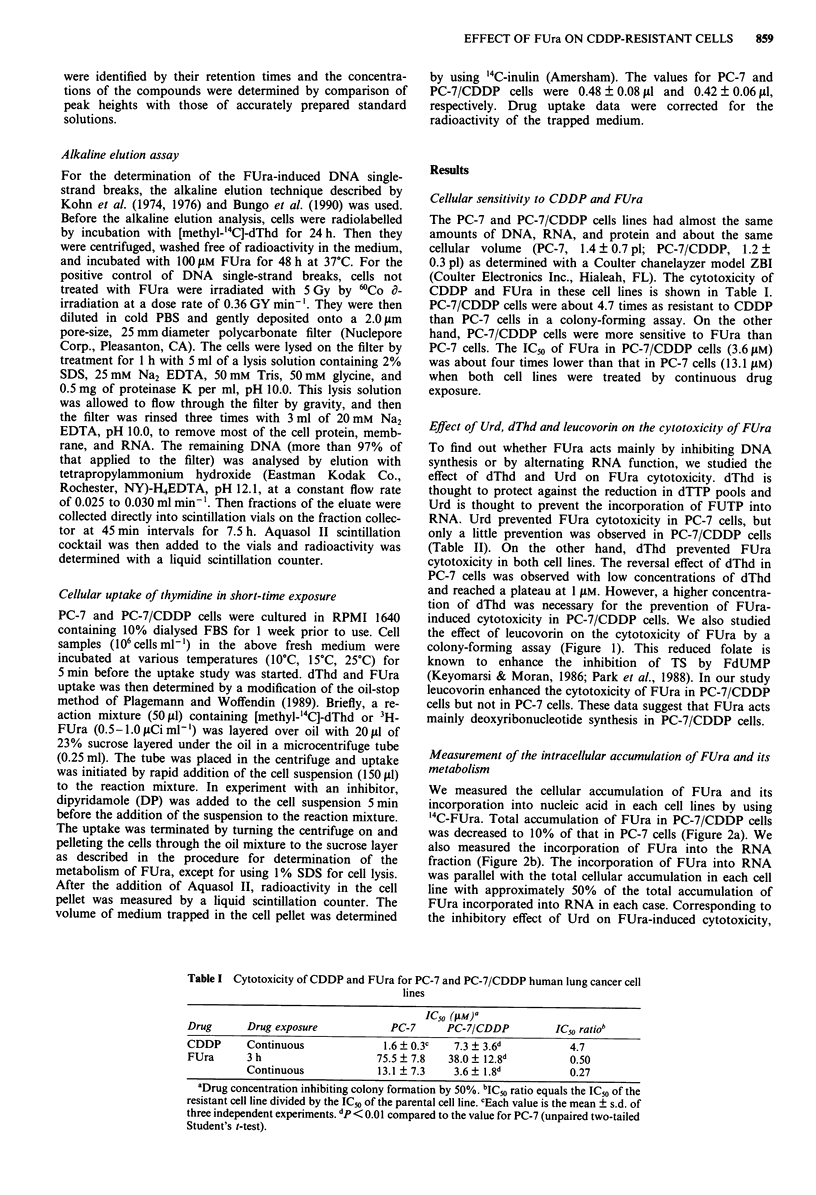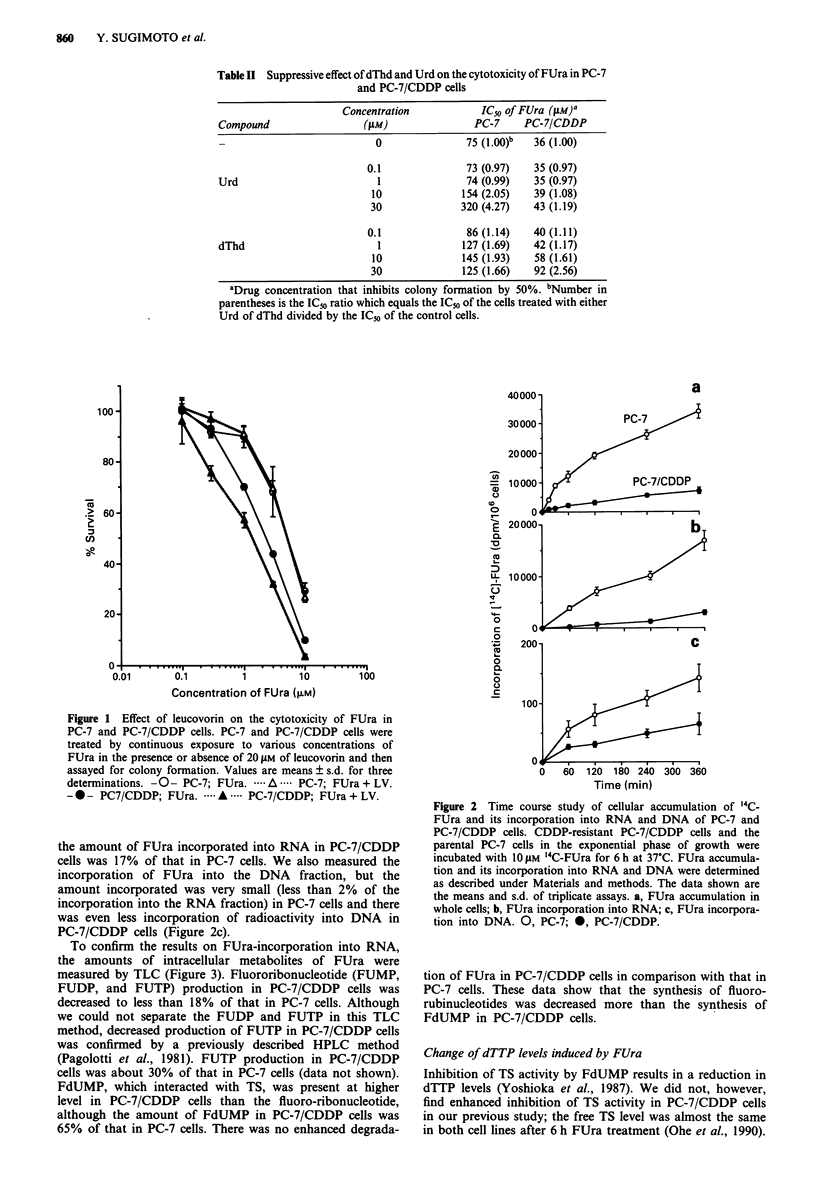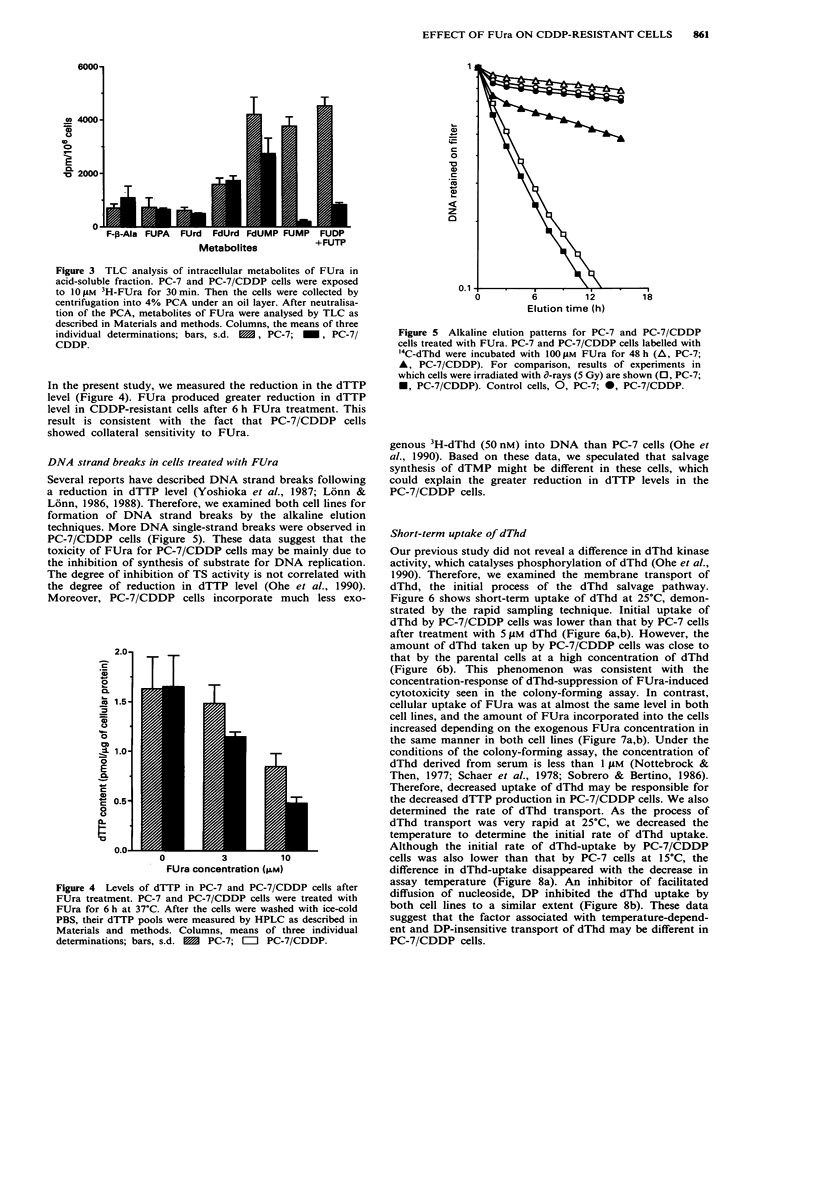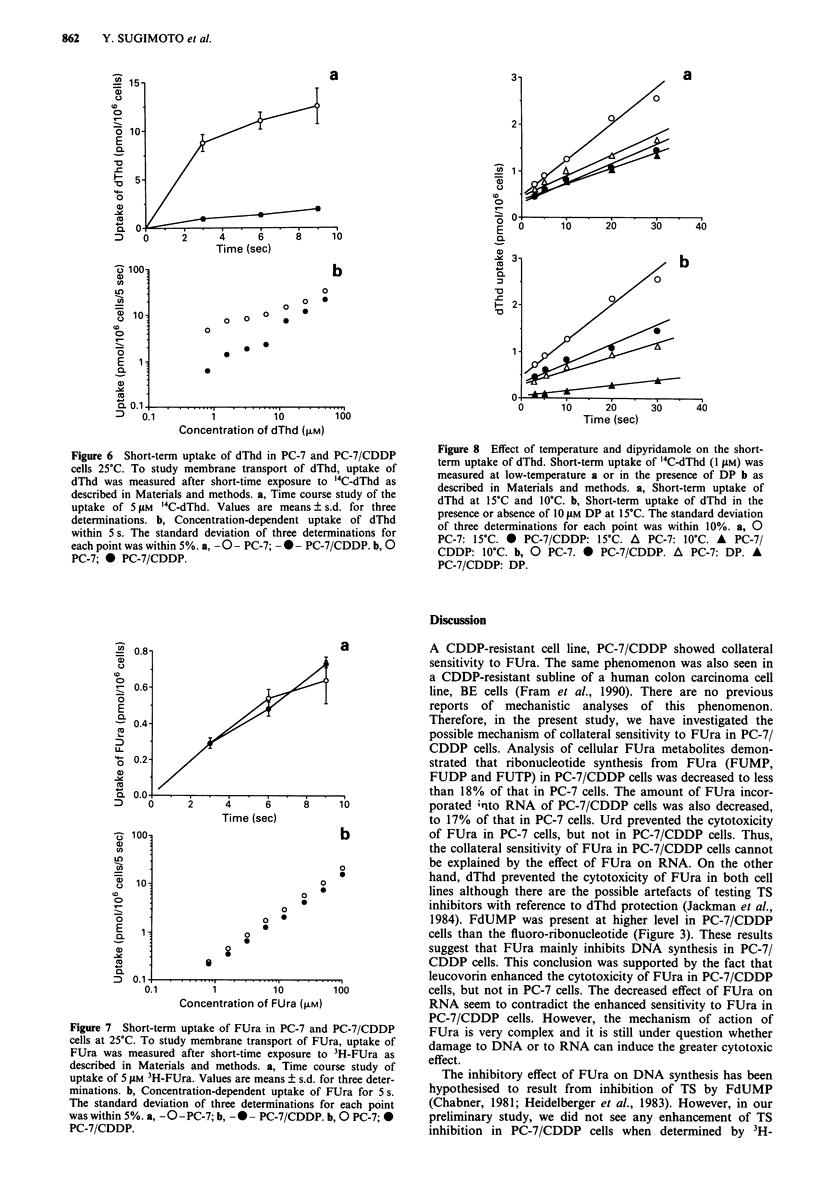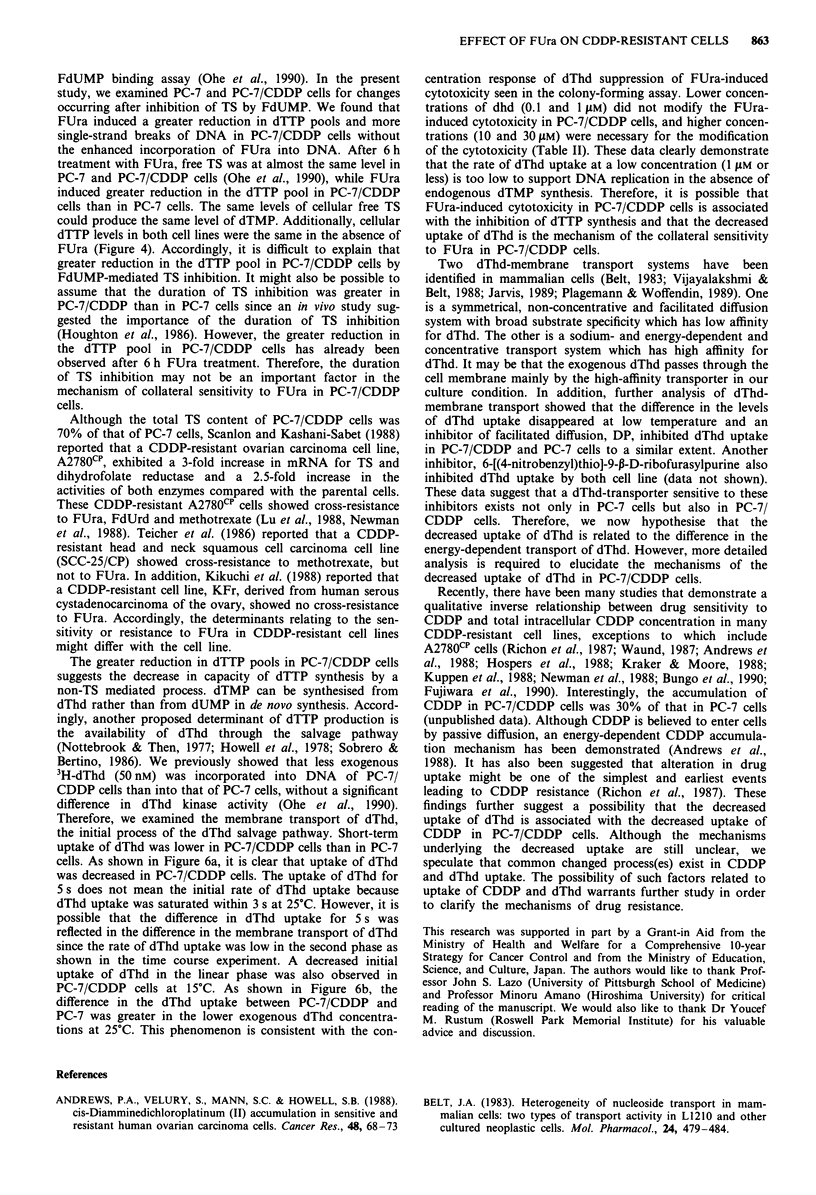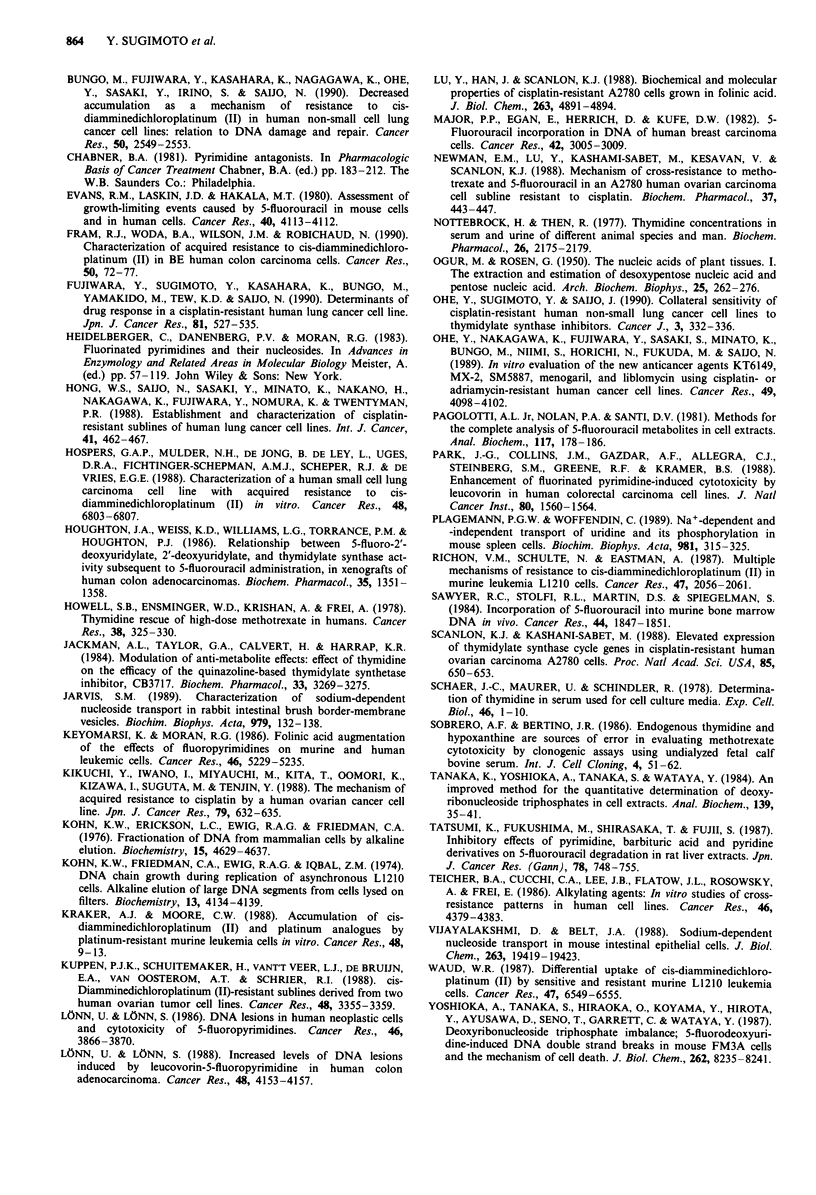# Mechanisms of collateral sensitivity to fluorouracil of a cis-diamminedichloroplatinum(II)-resistant human non-small lung cancer cell line.

**DOI:** 10.1038/bjc.1992.181

**Published:** 1992-06

**Authors:** Y. Sugimoto, Y. Ohe, K. Nishio, T. Ohmori, T. Morikage, Y. Fujiwara, N. Saijo

**Affiliations:** Pharmacology Division, National Cancer Center Research Institute, Tokyo, Japan.

## Abstract

A cisplatin(CDDP)-resistant subline of a human lung cancer cell line, PC-7/CDDP, was 4.7-fold more resistant to CDDP than the parent line in a colony-forming assay. The sensitivity of this cell line to anthracyclines, vinca-alkaloid, etoposide, mitomycin C, and bleomycin was similar to that of the parental line, PC-7. However, PC-7/CDDP exhibited 4-fold higher sensitivity to fluorouracil (FUra). Possible mechanisms associated with the collateral sensitivity to FUra were studied in PC-7/CDDP cells. The sensitivity of both cell lines to FUra did not correlate with the effect of FUra on RNA. On the other hand, FUra induced a greater reduction in dTTP pools and more single strand breaks in PC-7/CDDP than in PC-7 cells. These results suggest that the pathway for de novo deoxyribonucleotide synthesis may be a target for FUra in PC-7/CDDP cells. However, inhibition of thymidylate synthase after FUra treatment did not correlate with the DNA-directed activity of FUra. Based on the above findings, the decreased salvage synthesis of dTTP was considered a possible mechanism of the greater reduction of dTTP pools in PC-7/CDDP cells. However, the activity of dThd kinase was the same in both cell lines. In the presence of physiological concentrations of exogenous dThd in the serum, uptake of dThd was less in PC-7/CDDP cells than that in PC-7 cells. Our data suggest that FUra-induced cytotoxicity in PC-7/CDDP cells is associated with the inhibition of dTTP synthesis and that the decreased uptake of dThd is a possible mechanism of the collateral sensitivity to FUra in PC-7/CDDP cells.


Br. J. Cancer (1992), 65, 857–864 © Macmillan Press Ltd., 1992

### Mechanisms of collateral sensitivity to fluorouracil of a *cis*-diamminedichloroplatinum(II)-resistant human non-small lung cancer cell line

Y. Sugimoto[1,3], Y. Ohe[2], K. Nishio[1], T. Ohmori[1], T. Morikage[1], Y. Fujiwara[1] & N. Saijo[1]

[1]*Pharmacology Division, National Cancer Center Research Institute,* [2]*Department of Internal Medicine, National Cancer Center Hospital, 5-1-1 Tsukiji, Chuo-ku, Tokyo 104, Japan;* [3]*Cancer Research Laboratory, Hanno Research Center, Taiho Pharmaceutical Co, Ltd., 216-1 Nakayashita, yaoroshi, Hanno-city, Satima 357, Japan.*

**Summary** A cisplatin(CDDP)-resistant subline of a human lung cancer cell line, PC-7/CDDP, was 4.7-fold more resistant to CDDP than the parent line in a colony-forming assay. The sensitivity of this cell line to anthracyclines, vinca-alkaloid, etoposide, mitomycin C, and bleomycin was similar to that of the parental line, PC-7. However, PC-7/CDDP exhibited 4-fold higher sensitivity to fluorouracil (FUra). Possible mechanisms associated with the collateral sensitivity to FUra were studied in PC-7/CDDP cells. The sensitivity of both cell lines to FUra did not correlate with the effect of FUra on RNA. On the other hand, FUra induced a greater reduction in dTTP pools and more single strand breaks in PC-7/CDDP than in PC-7 cells. These results suggest that the pathway for *de novo* deoxyribonucleotide synthesis may be a target for FUra in PC-7/CDDP cells. However, inhibition of thymidylate synthase after FUra treatment did not correlate with the DNA-directed activity of FUra. Based on the above findings, the decreased salvage synthesis of dTTP was considered a possible mechanism of the greater reduction of dTTP pools in PC-7/CDDP cells. However, the activity of dThd kinase was the same in both cell lines. In the presence of physiological concentrations of exogenous dThd in the serum, uptake of dThd was less in PC-7/CDDP cells than that in PC-7 cells. Our data suggest that FUra-induced cytotoxicity in PC-7/CDDP cells is associated with the inhibition of dTTP synthesis and that the decreased uptake of dThd is a possible mechanism of the collateral sensitivity to FUra in PC-7/CDDP cells.

CDDP is one of the most effective antitumour drugs available for the clinical treatment of human cancers. However, tumours become clinically unresponsive to this drug upon continued treatment. Therefore, it is important to elucidate the mechanisms or related processes of resistance to CDDP. We previously established a CDDP-resistant subline, PC-7/CDDP, from a human lung cancer cell line, PC-7, in order to elucidate the mechanisms of resistance to CDDP and to assist in developing strategies to circumvent CDDP resistance (Hong *et al.*, 1988; Bungo *et al.*, 1990; Fujiwara *et al.*, 1990). We demonstrated that PC-7/CDDP cells do not exhibit cross-resistance or collateral sensitivity to adriamycin, daunomycin, mitomycin C, bleomycin, etoposide, or vindesine. In contrast, PC-7/CDDP cells exhibited collateral sensitivity to FUra (Ohe *et al.*, 1990). Determination of the mechanisms of such enhanced drug sensitivity may reveal the factors that contribute to cellular sensitivity to FUra and their relation to the mechanisms of CDDP resistance. Therefore, we searched for the possible mechanisms associated with the collateral sensitivity to FUra in PC-7/CDDP cells.

The mechanism of action of FUra is complex and cytotoxicity appears to depend upon the cell type being studied. FUra is known to exert its cytotoxic effect by at least two mechanisms: incorporation of FUra into RNA and inhibition of thymidylate synthase (TS) (Chabner, 1981; Heidelberger *et al.*, 1983). FUra is anabolised to the ribonucleotide, FUTP. FUTP is incorporated into RNA, which may alter RNA function. FUra is also anabolised to the deoxyribomonophosphate, FdUMP. In the presence of methylenetetrahydrofolate cofactor, FdUMP forms a covalent ternary complex with TS, inhibiting the *de novo* synthesis of dTMP. Depletion of dTMP and then dTTP interferes with DNA synthesis and induces DNA strand breaks (Pagolotti *et al.*, 1981; Lonn & Lonn, 1986; Yoshioka *et al.*, 1987). In addition, it has been shown that FUra can be incorporated into DNA, and this may also contribute to its cytotoxicity (Major *et al.*, 1982; Sawyer *et al.*, 1984). Furthermore, FUra can be catabolised to $H_2$FUra and consequently to FUPA and further to F-β-Ala.

We previously tried to determine the contribution of TS inhibition by FUra in PC-7/CDDP cells (Ohe *et al.*, 1990). However, there was no correlation between the level of TS inhibition revealed by $^3$H-FdUMP binding assay and sensitivity to FUra. These data suggest that the factors responsible for TS inhibition do not contribute to the collateral sensitivity of FUra. Therefore, we have examined the effects of FUra on RNA and dTTP pools to investigate further the underlying mechanisms responsible for the collateral sensitivity to FUra in PC-7/CDDP cells.

### Materials and methods

### *Drugs and chemicals*

RPMI 1640 medium and PBS were purchased from Nissui Pharmaceutical Co., Tokyo, Japan. Other agents were obtained from the following sources: CDDP from Bristol Myers Squibb Co., Tokyo, Japan; FUra from Kyowa Hakko Kogyo Co., Ltd, Tokyo; FUrd from Mitsui Pharmaceutical Inc., Tokyo; [6-$^3$H]-FUra (specific activity, 19.3 Ci mmol$^{-1}$) and [methyl-$^{14}$C]-dThd (specific activity, 60 mCi mmol$^{-1}$) from Amersham Japan Ltd, Tokyo; [6-$^{14}$C]-FUra (specific activity, 56 mCi mmol$^{-1}$) from New England Nuclear Corp., Boston, MA. All other drugs and chemicals were purchased from Sigma Chemical Co., St Louis, MO if not otherwise reported.

### *Cell lines and culture*

Line PC-7 derived from an adenocarcinoma of the lung with no prior exposure to chemotherapeutic agents was kindly donated by Professor Y. Hayata of the Tokyo Medical College. The CDDP-resistant cell line, PC-7/CDDP, was established in our laboratory by exposing parental cells to stepwise increased concentrations of CDDP and cloned by the

Correspondence: N. Saijo.
Received 23 December 1991; and in revised form 21 February 1992.

limiting-dilution technique (Hong et al., 1988; Ohe et al., 1990). Resistance to CDDP was stable for at least 6 months in CDDP-free medium. Prior to use for experiments, the cell lines were propagated by culturing them in RPMI 1640 medium supplemented with 10% heat-inactivated FBS (Immuno-Biochemical Laboratories, Fujioka, Japan), penicillin (100 units $ml^{-1}$) and streptomycin (100 $\mu g\ ml^{-1}$). PC-7 and PC-7/CDDP cells were grown as stationary suspension cultures. Cell cultures were initiated by seeding the medium with approximately $5 \times 10^4$ cells $ml^{-1}$ and grew exponentially to at least $8 \times 10^5$ cells $ml^{-1}$. All experiments were performed with cells in the exponential phase of growth. The PC-7/CDDP were cultured for more than 2 weeks before analysis in CDDP-free medium. These cells were found to be free of mycoplasmal contamination by examination with a Hoeschst stain kit for detection of *Mycoplasma* in cell culture (Flow Laboratories, Inc., McLean, VA).

*Colony-forming assay*

Colony-forming efficiency was assessed in a double-layer soft agar system as previously described (Ohe et al., 1989, 1990), except for the use of the same culture medium in both top and bottom layers. PC-7 and PC-7/CDDP were plated at a concentration of $3 \times 10^4$ cells/well with various concentrations of FUra. The culture plates were incubated at 37°C in humidified air with 5% $CO_2$. After continuous exposure to FUra for 15–20 days, colonies larger than 50 $\mu m$ in diameter were counted with an automatic particle counter (CP-2000, Shiraimatsu Co., Ltd, Tokyo). All assays were performed in triplicate. The plating efficiency of cell lines without the drug was 8–9%. The effect of leucovorin (5-formyl-tetrahydrofolate) on the cytotoxicity of FUra was determined by the same procedure as described above, except for the addition of 1-leucovorin [Lederle (Japan), Ltd, Tokyo]. In the experiments on the effect of Urd or dThd on FUra cytotoxicity, each cell line was cultured for 3 days in RPMI 1640 supplemented with dialysed FBS (Cell Culture Laboratories, Cleveland, OH) in 6-well culture plates. Cells were then treated with FUra at various concentrations for 3 h in a $CO_2$ incubator. FUra-treated cells were washed with RPMI 1640 medium and cultured in soft agar with or without various concentrations of dThd and Urd in RPMI 1640 medium plus 10% dialysed FBS as described above. All drugs were dissolved in distilled water and stored at − 20°C. They were diluted with culture medium just before each experiment.

*Cellular accumulation and intracellular metabolism of FUra*

Suspensions of PC-7 and PC-7/CDDP cells ($2 \times 10^8$ cells in total, respectively) were adjusted to a density of $3 \times 10^5$ cells/$ml^{-1}$ and then incubated at 37°C in a $CO_2$ incubator. Experiments on total cellular accumulation of FUra and its incorporation into RNA and DNA were initiated by rapid addition of a small volume of a solution of $^{14}$C-FUra. The final concentration of $^{14}$C-FUra was 10 $\mu$M. At the time indicated, the cell suspension was washed three times with ice-cold PBS by centrifugation at 250 *g* for 5 min, and the cells were lysed in 10 mM Tris-HCl buffer, pH 8.0, containing 1 mM EDTA and 0.1% SDS. Radioactivity in the cells was counted with 10 ml of Aquasol II scintillation cocktail (New England Nuclear) in a liquid scintillation counter LS3801 [Beckman Instruments (Japan), Tokyo]. Aliquots of the lysates were precipitated with 4% PCA. $^{14}$C-FUra incorporated into RNA was considered to be originally acid-precipitable material that was no longer precipitable after incubation for 20 h in 0.1 N KOH at 37°C, a condition which degrades RNA to acid-soluble material. After KOH treatment, the solution was neutralised with 0.1 N HCl and reprecipitated by addition of ice-cold 4% PCA. After centrifugation at 1,600 *g* for 10 min, the radioactivity of the supernatant was counted in Aquasol II. To measure the incorporation of $^{14}$C-FUra into DNA, the remaining precipitate was washed twice with ice-cold 4% PCA and boiled in 4% PCA for 20 min, and radioactivity incorporated in this fraction was counted. The RNA and DNA content of these alkali-hydrolysed fractions was determined by UV absorption with hydrolysates of yeast RNA and calf thymus DNA as standards (Ogur & Rosen, 1950).

Intracellular metabolism of FUra was determined by the same initial procedure except for the use of $^3$H-FUra instead of $^{14}$C-FUra. As FdUMP has been reported to be rapidly degraded (Evans et al., 1980), we used the method of rapid cell-separation from the culture medium to prevent the intracellular degradation of FUra metabolites. After incubation for 30 min, 4 ml of the cell suspension was layered over 1 ml of oil [1.1:1 mixture of dibutyl phthalate and dioctyl phthalate oils (Tokyo-Kasei Co., Ltd, Tokyo)] with 100 $\mu$l of 4% PCA layered under the oil. The tubes were immediately centrifuged at 1,600 *g* for 3 min and a portion of the acid layer was removed and neutralised with 1.5 N KOH and stored at − 70°C until use. Fifteen microlitres of the neutralised supernatant was spotted on a TLC plate with unlabelled markers. Cellular fluoronucleotides (FdUMP, FUMP, FUDP and FUTP) were separated on polyethleneimine-cellulose plates (Merck Japan Ltd, Tokyo) by using 2% boric acid: 2 M NaCl (70:35) as a developer. Rf values of reference compounds were as follows: FdUrd and FUrd, 0.84; FUra, 0.75; FdUMP, 0.71; FUMP, 0.55; ureidopropionic acid, 0.88; FUDP, 0.12; FUTP, 0.04. Fluoronucleoside, base and other degradation products of FUra, were detected by silica gel $60F_{254}$ plates (Merck) with chloroform: methanol:acetic acid (80:20:5) used as a developer (Tatsumi et al., 1987). Rf values of reference compounds were as follows: FUra, 0.62; FdUrd, 0.52; FUrd, 0.33; FdUMP and FUMP, 0.00; FUPA, 0.43; F-$\beta$-Ala, 0.03. Rf values of FUDP and FUTP were determined by fluorography of metabolites in cells or liver using E$^3$HANCE spray (New England Nuclear). Spots of unlabelled markers were then scraped out and placed in scintillation vials. Radioactivity was eluted from the solid support by shaking with 4% PCA. Aquasol II scintillation cocktail was then added to the vials and radioactivity was measured.

*Measurement of intracellular dTTP pool by HPLC*

Intracellular dTTP levels in PC-7 and PC-7/CDDP were measured by the method of Tanaka et al. (1984). In brief, the cells ($1-2 \times 10^5\ ml^{-1}$) in RPMI 1640 medium plus 10% FBS were incubated in a $CO_2$ incubator with and without FUra. Six hours after the addition of FUra, 25 ml of cell suspension was transferred to a 50 ml tube, 5 ml of ice-cold PBS was added and then suspension was centrifuged at 250 *g* for 5 min. The cell pellet was washed with 30 ml of ice-cold PBS by centrifugation as above. The volume of the cell suspension was adjusted to 200 $\mu$l by the addition of PBS to the cell pellet on ice. One hundred and fifty microlitres of the cell suspension was transferred to a 1.5 ml tube and the cell suspension remaining in the 50 ml tube was used for protein assay (BCA protein assay kit, Pierce Chemical Co., Rockford, IL) with bovine serum albumin as standard. Then 7.5 $\mu$l of ice-cold 100% TCA was added to the 150 $\mu$l cell suspension. The sample was then vortexed, placed on ice for 10 min and were centrifuged at 17,000 *g* for 20 s. The acid-soluble extract was neutralised by extraction with two volumes of 0.5 M tri-n-octylamine in trichlorotrifluoroethane. For deoxyribonucleoside triphosphate determination, ribonucleotides were destroyed by the action of periodate and methylamine as follows: After addition of 20 $\mu$l or 20 mM deoxyguanosine to 80 $\mu$l of neutralised cell extract, 20 $\mu$l of 0.2 M PCA was added, followed by 30 $\mu$l of 4 M methylamine phosphate, pH 7.5. After incubation for 30 min at 37°C, 2 $\mu$l of 1 M rhamnose was added to destroy the remaining periodate. A Partisil-10 SAX anion exchange column (4.6 × 250 mm, Whatman Inc., Clifton, NJ) was used for HPLC and 0.4 M ammonium dishydrogenphosphate:acetonitrorile, 10:1 (v/v), pH 3.30 was used as elutant with a flow rate of 1.5 ml $min^{-1}$. A 100 $\mu$l aliquot of the periodate-treated sample was injected into the column at ambient temperature, and the column eluate was monitored simultaneously at 254 nm. Compounds were identified by their retention times and the concentrations of the compounds were determined by comparison of peak heights with those of accurately prepared standard solutions.

*Alkaline elution assay*

For the determination of the FUra-induced DNA single-strand breaks, the alkaline elution technique described by Kohn *et al.* (1974, 1976) and Bungo *et al.* (1990) was used. Before the alkaline elution analysis, cells were radiolabelled by incubation with [methyl-$^{14}$C]-dThd for 24 h. Then they were centrifuged, washed free of radioactivity in the medium, and incubated with 100 μM FUra for 48 h at 37°C. For the positive control of DNA single-strand breaks, cells not treated with FUra were irradiated with 5 Gy by $^{60}$Co ∂-irradiation at a dose rate of 0.36 GY min$^{-1}$. They were then diluted in cold PBS and gently deposited onto a 2.0 μm pore-size, 25 mm diameter polycarbonate filter (Nuclepore Corp., Pleasanton, CA). The cells were lysed on the filter by treatment for 1 h with 5 ml of a lysis solution containing 2% SDS, 25 mM $Na_2$ EDTA, 50 mM Tris, 50 mM glycine, and 0.5 mg of proteinase K per ml, pH 10.0. This lysis solution was allowed to flow through the filter by gravity, and then the filter was rinsed three times with 3 ml of 20 mM $Na_2$ EDTA, pH 10.0, to remove most of the cell protein, membrane, and RNA. The remaining DNA (more than 97% of that applied to the filter) was analysed by elution with tetrapropylammonium hydroxide (Eastman Kodak Co., Rochester, NY)-$H_4$EDTA, pH 12.1, at a constant flow rate of 0.025 to 0.030 ml min$^{-1}$. Then fractions of the eluate were collected directly into scintillation vials on the fraction collector at 45 min intervals for 7.5 h. Aquasol II scintillation cocktail was then added to the vials and radioactivity was determined with a liquid scintillation counter.

*Cellular uptake of thymidine in short-time exposure*

PC-7 and PC-7/CDDP cells were cultured in RPMI 1640 containing 10% dialysed FBS for 1 week prior to use. Cell samples ($10^6$ cells ml$^{-1}$) in the above fresh medium were incubated at various temperatures (10°C, 15°C, 25°C) for 5 min before the uptake study was started. dThd and FUra uptake was then determined by a modification of the oil-stop method of Plagemann and Woffendin (1989). Briefly, a reaction mixture (50 μl) containing [methyl-$^{14}$C]-dThd or $^3$H-FUra (0.5–1.0 μCi ml$^{-1}$) was layered over oil with 20 μl of 23% sucrose layered under the oil in a microcentrifuge tube (0.25 ml). The tube was placed in the centrifuge and uptake was initiated by rapid addition of the cell suspension (150 μl) to the reaction mixture. In experiment with an inhibitor, dipyridamole (DP) was added to the cell suspension 5 min before the addition of the suspension to the reaction mixture. The uptake was terminated by turning the centrifuge on and pelleting the cells through the oil mixture to the sucrose layer as described in the procedure for determination of the metabolism of FUra, except for using 1% SDS for cell lysis. After the addition of Aquasol II, radioactivity in the cell pellet was measured by a liquid scintillation counter. The volume of medium trapped in the cell pellet was determined by using $^{14}$C-inulin (Amersham). The values for PC-7 and PC-7/CDDP cells were 0.48 ± 0.08 μl and 0.42 ± 0.06 μl, respectively. Drug uptake data were corrected for the radioactivity of the trapped medium.

### Results

*Cellular sensitivity to CDDP and FUra*

The PC-7 and PC-7/CDDP cells lines had almost the same amounts of DNA, RNA, and protein and about the same cellular volume (PC-7, 1.4 ± 0.7 pl; PC-7/CDDP, 1.2 ± 0.3 pl) as determined with a Coulter chanelayzer model ZBI (Coulter Electronics Inc., Hialeah, FL). The cytotoxicity of CDDP and FUra in these cell lines is shown in Table I. PC-7/CDDP cells were about 4.7 times as resistant to CDDP than PC-7 cells in a colony-forming assay. On the other hand, PC-7/CDDP cells were more sensitive to FUra than PC-7 cells. The $IC_{50}$ of FUra in PC-7/CDDP cells (3.6 μM) was about four times lower than that in PC-7 cells (13.1 μM) when both cell lines were treated by continuous drug exposure.

*Effect of Urd, dThd and leucovorin on the cytotoxicity of FUra*

To find out whether FUra acts mainly by inhibiting DNA synthesis or by alternating RNA function, we studied the effect of dThd and Urd on FUra cytotoxicity. dThd is thought to protect against the reduction in dTTP pools and Urd is thought to prevent the incorporation of FUTP into RNA. Urd prevented FUra cytotoxicity in PC-7 cells, but only a little prevention was observed in PC-7/CDDP cells (Table II). On the other hand, dThd prevented FUra cytotoxicity in both cell lines. The reversal effect of dThd in PC-7 cells was observed with low concentrations of dThd and reached a plateau at 1 μM. However, a higher concentration of dThd was necessary for the prevention of FUra-induced cytotoxicity in PC-7/CDDP cells. We also studied the effect of leucovorin on the cytotoxicity of FUra by a colony-forming assay (Figure 1). This reduced folate is known to enhance the inhibition of TS by FdUMP (Keyomarsi & Moran, 1986; Park *et al.*, 1988). In our study leucovorin enhanced the cytotoxicity of FUra in PC-7/CDDP cells but not in PC-7 cells. These data suggest that FUra acts mainly deoxyribonucleotide synthesis in PC-7/CDDP cells.

*Measurement of the intracellular accumulation of FUra and its metabolism*

We measured the cellular accumulation of FUra and its incorporation into nucleic acid in each cell lines by using $^{14}$C-FUra. Total accumulation of FUra in PC-7/CDDP cells was decreased to 10% of that in PC-7 cells (Figure 2a). We also measured the incorporation of FUra into the RNA fraction (Figure 2b). The incorporation of FUra into RNA was parallel with the total cellular accumulation in each cell line with approximately 50% of the total accumulation of FUra incorporated into RNA in each case. Corresponding to the inhibitory effect of Urd on FUra-induced cytotoxicity,

**Table I** Cytotoxicity of CDDP and FUra for PC-7 and PC-7/CDDP human lung cancer cell lines

| Drug | Drug exposure | $IC_{50}$ (μM)[a] PC-7 | PC-7/CDDP | $IC_{50}$ ratio[b] |
|---|---|---|---|---|
| CDDP | Continuous | 1.6 ± 0.3[c] | 7.3 ± 3.6[d] | 4.7 |
| FUra | 3 h | 75.5 ± 7.8 | 38.0 ± 12.8[d] | 0.50 |
| | Continuous | 13.1 ± 7.3 | 3.6 ± 1.8[d] | 0.27 |

[a]Drug concentration inhibiting colony formation by 50%. [b]$IC_{50}$ ratio equals the $IC_{50}$ of the resistant cell line divided by the $IC_{50}$ of the parental cell line. [c]Each value is the mean ± s.d. of three independent experiments. [d]$P < 0.01$ compared to the value for PC-7 (unpaired two-tailed Student's *t*-test).

**Table II** Suppressive effect of dThd and Urd on the cytotoxicity of FUra in PC-7 and PC-7/CDDP cells

| *Compound* | *Concentration (μM)* | *$IC_{50}$ of FUra (μM)[a]* *PC-7* | *PC-7/CDDP* |
|---|---|---|---|
| – | 0 | 75 (1.00)[b] | 36 (1.00) |
| Urd | 0.1 | 73 (0.97) | 35 (0.97) |
| | 1 | 74 (0.99) | 35 (0.97) |
| | 10 | 154 (2.05) | 39 (1.08) |
| | 30 | 320 (4.27) | 43 (1.19) |
| dThd | 0.1 | 86 (1.14) | 40 (1.11) |
| | 1 | 127 (1.69) | 42 (1.17) |
| | 10 | 145 (1.93) | 58 (1.61) |
| | 30 | 125 (1.66) | 92 (2.56) |

[a]Drug concentration that inhibits colony formation by 50%. [b]Number in parentheses is the $IC_{50}$ ratio which equals the $IC_{50}$ of the cells treated with either Urd of dThd divided by the $IC_{50}$ of the control cells.

**Figure 1** Effect of leucovorin on the cytotoxicity of FUra in PC-7 and PC-7/CDDP cells. PC-7 and PC-7/CDDP cells were treated by continuous exposure to various concentrations of FUra in the presence or absence of 20 μM of leucovorin and then assayed for colony formation. Values are means ± s.d. for three determinations. –O– PC-7; FUra. ····Δ···· PC-7; FUra + LV. –●– PC7/CDDP; FUra. ····▲···· PC-7/CDDP; FUra + LV.

**Figure 2** Time course study of cellular accumulation of [14]C-FUra and its incorporation into RNA and DNA of PC-7 and PC-7/CDDP cells. CDDP-resistant PC-7/CDDP cells and the parental PC-7 cells in the exponential phase of growth were incubated with 10 μM [14]C-FUra for 6 h at 37°C. FUra accumulation and its incorporation into RNA and DNA were determined as described under Materials and methods. The data shown are the means and s.d. of triplicate assays. **a**, FUra accumulation in whole cells; **b**, FUra incorporation into RNA; **c**, FUra incorporation into DNA. O, PC-7; ●, PC-7/CDDP.

the amount of FUra incorporated into RNA in PC-7/CDDP cells was 17% of that in PC-7 cells. We also measured the incorporation of FUra into the DNA fraction, but the amount incorporated was very small (less than 2% of the incorporation into the RNA fraction) in PC-7 cells and there was even less incorporation of radioactivity into DNA in PC-7/CDDP cells (Figure 2c).

To confirm the results on FUra-incorporation into RNA, the amounts of intracellular metabolites of FUra were measured by TLC (Figure 3). Fluororibonucleotide (FUMP, FUDP, and FUTP) production in PC-7/CDDP cells was decreased to less than 18% of that in PC-7 cells. Although we could not separate the FUDP and FUTP in this TLC method, decreased production of FUTP in PC-7/CDDP cells was confirmed by a previously described HPLC method (Pagolotti *et al.*, 1981). FUTP production in PC-7/CDDP cells was about 30% of that in PC-7 cells (data not shown). FdUMP, which interacted with TS, was present at higher level in PC-7/CDDP cells than the fluoro-ribonucleotide, although the amount of FdUMP in PC-7/CDDP cells was 65% of that in PC-7 cells. There was no enhanced degradation of FUra in PC-7/CDDP cells in comparison with that in PC-7 cells. These data show that the synthesis of fluororubinucleotides was decreased more than the synthesis of FdUMP in PC-7/CDDP cells.

### *Change of dTTP levels induced by FUra*

Inhibition of TS activity by FdUMP results in a reduction in dTTP levels (Yoshioka *et al.*, 1987). We did not, however, find enhanced inhibition of TS activity in PC-7/CDDP cells in our previous study; the free TS level was almost the same in both cell lines after 6 h FUra treatment (Ohe *et al.*, 1990).

**Figure 3** TLC analysis of intracellular metabolites of FUra in acid-soluble fraction. PC-7 and PC-7/CDDP cells were exposed to 10 $\mu$M $^3$H-FUra for 30 min. Then the cells were collected by centrifugation into 4% PCA under an oil layer. After neutralisation of the PCA, metabolites of FUra were analysed by TLC as described in Materials and methods. Columns, the means of three individual determinations; bars, s.d. ▨, PC-7; ■, PC-7/CDDP.

In the present study, we measured the reduction in the dTTP level (Figure 4). FUra produced greater reduction in dTTP level in CDDP-resistant cells after 6 h FUra treatment. This result is consistent with the fact that PC-7/CDDP cells showed collateral sensitivity to FUra.

*DNA strand breaks in cells treated with FUra*

Several reports have described DNA strand breaks following a reduction in dTTP level (Yoshioka *et al.*, 1987; Lönn & Lönn, 1986, 1988). Therefore, we examined both cell lines for formation of DNA strand breaks by the alkaline elution techniques. More DNA single-strand breaks were observed in PC-7/CDDP cells (Figure 5). These data suggest that the toxicity of FUra for PC-7/CDDP cells may be mainly due to the inhibition of synthesis of substrate for DNA replication. The degree of inhibition of TS activity is not correlated with the degree of reduction in dTTP level (Ohe *et al.*, 1990). Moreover, PC-7/CDDP cells incorporate much less exogenous $^3$H-dThd (50 nM) into DNA than PC-7 cells (Ohe *et al.*, 1990). Based on these data, we speculated that salvage synthesis of dTMP might be different in these cells, which could explain the greater reduction in dTTP levels in the PC-7/CDDP cells.

**Figure 5** Alkaline elution patterns for PC-7 and PC-7/CDDP cells treated with FUra. PC-7 and PC-7/CDDP cells labelled with $^{14}$C-dThd were incubated with 100 $\mu$M FUra for 48 h (△, PC-7; ▲, PC-7/CDDP). For comparison, results of experiments in which cells were irradiated with ∂-rays (5 Gy) are shown (□, PC-7; ■, PC-7/CDDP). Control cells, ○, PC-7; ●, PC-7/CDDP.

**Figure 4** Levels of dTTP in PC-7 and PC-7/CDDP cells after FUra treatment. PC-7 and PC-7/CDDP cells were treated with FUra for 6 h at 37°C. After the cells were washed with ice-cold PBS, their dTTP pools were measured by HPLC as described in Materials and methods. Columns, means of three individual determinations; bars, s.d. ▨ PC-7; ▭ PC-7/CDDP.

*Short-term uptake of dThd*

Our previous study did not reveal a difference in dThd kinase activity, which catalyses phosphorylation of dThd (Ohe *et al.*, 1990). Therefore, we examined the membrane transport of dThd, the initial process of the dThd salvage pathway. Figure 6 shows short-term uptake of dThd at 25°C, demonstrated by the rapid sampling technique. Initial uptake of dThd by PC-7/CDDP cells was lower than that by PC-7 cells after treatment with 5 $\mu$M dThd (Figure 6a,b). However, the amount of dThd taken up by PC-7/CDDP cells was close to that by the parental cells at a high concentration of dThd (Figure 6b). This phenomenon was consistent with the concentration-response of dThd-suppression of FUra-induced cytotoxicity seen in the colony-forming assay. In contrast, cellular uptake of FUra was at almost the same level in both cell lines, and the amount of FUra incorporated into the cells increased depending on the exogenous FUra concentration in the same manner in both cell lines (Figure 7a,b). Under the conditions of the colony-forming assay, the concentration of dThd derived from serum is less than 1 $\mu$M (Nottebrock & Then, 1977; Schaer *et al.*, 1978; Sobrero & Bertino, 1986). Therefore, decreased uptake of dThd may be responsible for the decreased dTTP production in PC-7/CDDP cells. We also determined the rate of dThd transport. As the process of dThd transport was very rapid at 25°C, we decreased the temperature to determine the initial rate of dThd uptake. Although the initial rate of dThd-uptake by PC-7/CDDP cells was also lower than that by PC-7 cells at 15°C, the difference in dThd-uptake disappeared with the decrease in assay temperature (Figure 8a). An inhibitor of facilitated diffusion of nucleoside, DP inhibited the dThd uptake by both cell lines to a similar extent (Figure 8b). These data suggest that the factor associated with temperature-dependent and DP-insensitive transport of dThd may be different in PC-7/CDDP cells.

**Figure 6** Short-term uptake of dThd in PC-7 and PC-7/CDDP cells 25°C. To study membrane transport of dThd, uptake of dThd was measured after short-time exposure to $^{14}$C-dThd as described in Materials and methods. **a**, Time course study of the uptake of 5 μM $^{14}$C-dThd. Values are means ± s.d. for three determinations. **b**, Concentration-dependent uptake of dThd within 5 s. The standard deviation of three determinations for each point was within 5%. **a**, -O- PC-7; -●- PC-7/CDDP. **b**, O PC-7; ● PC-7/CDDP.

**Figure 8** Effect of temperature and dipyridamole on the short-term uptake of dThd. Short-term uptake of $^{14}$C-dThd (1 μM) was measured at low-temperature **a** or in the presence of DP **b** as described in Materials and methods. **a**, Short-term uptake of dThd at 15°C and 10°C. **b**, Short-term uptake of dThd in the presence or absence of 10 μM DP at 15°C. The standard deviation of three determinations for each point was within 10%. **a**, O PC-7: 15°C. ● PC-7/CDDP: 15°C. Δ PC-7: 10°C. ▲ PC-7/CDDP: 10°C. **b**, O PC-7. ● PC-7/CDDP. Δ PC-7: DP. ▲ PC-7/CDDP: DP.

**Figure 7** Short-term uptake of FUra in PC-7 and PC-7/CDDP cells at 25°C. To study membrane transport of FUra, uptake of FUra was measured after short-time exposure to $^3$H-FUra as described in Materials and methods. **a**, Time course study of uptake of 5 μM $^3$H-FUra. Values are means ± s.d. for three determinations. **b**, Concentration-dependent uptake of FUra for 5 s. The standard deviation of three determinations for each point was within 5%. **a**, -O-PC-7; **b**, -●- PC-7/CDDP. **b**, O PC-7; ● PC-7/CDDP.

### Discussion

A CDDP-resistant cell line, PC-7/CDDP showed collateral sensitivity to FUra. The same phenomenon was also seen in a CDDP-resistant subline of a human colon carcinoma cell line, BE cells (Fram et al., 1990). There are no previous reports of mechanistic analyses of this phenomenon. Therefore, in the present study, we have investigated the possible mechanism of collateral sensitivity to FUra in PC-7/CDDP cells. Analysis of cellular FUra metabolites demonstrated that ribonucleotide synthesis from FUra (FUMP, FUDP and FUTP) in PC-7/CDDP cells was decreased to less than 18% of that in PC-7 cells. The amount of FUra incorporated into RNA of PC-7/CDDP cells was also decreased, to 17% of that in PC-7 cells. Urd prevented the cytotoxicity of FUra in PC-7 cells, but not in PC-7/CDDP cells. Thus, the collateral sensitivity of FUra in PC-7/CDDP cells cannot be explained by the effect of FUra on RNA. On the other hand, dThd prevented the cytotoxicity of FUra in both cell lines although there are the possible artefacts of testing TS inhibitors with reference to dThd protection (Jackman et al., 1984). FdUMP was present at higher level in PC-7/CDDP cells than the fluoro-ribonucleotide (Figure 3). These results suggest that FUra mainly inhibits DNA synthesis in PC-7/CDDP cells. This conclusion was supported by the fact that leucovorin enhanced the cytotoxicity of FUra in PC-7/CDDP cells, but not in PC-7 cells. The decreased effect of FUra on RNA seem to contradict the enhanced sensitivity to FUra in PC-7/CDDP cells. However, the mechanism of action of FUra is very complex and it is still under question whether damage to DNA or to RNA can induce the greater cytotoxic effect.

The inhibitory effect of FUra on DNA synthesis has been hypothesised to result from inhibition of TS by FdUMP (Chabner, 1981; Heidelberger et al., 1983). However, in our preliminary study, we did not see any enhancement of TS inhibition in PC-7/CDDP cells when determined by $^3$H-

FdUMP binding assay (Ohe *et al.*, 1990). In the present study, we examined PC-7 and PC-7/CDDP cells for changes occurring after inhibition of TS by FdUMP. We found that FUra induced a greater reduction in dTTP pools and more single-strand breaks of DNA in PC-7/CDDP cells without the enhanced incorporation of FUra into DNA. After 6 h treatment with FUra, free TS was at almost the same level in PC-7 and PC-7/CDDP cells (Ohe *et al.*, 1990), while FUra induced greater reduction in the dTTP pool in PC-7/CDDP cells than in PC-7 cells. The same levels of cellular free TS could produce the same level of dTMP. Additionally, cellular dTTP levels in both cell lines were the same in the absence of FUra (Figure 4). Accordingly, it is difficult to explain that greater reduction in the dTTP pool in PC-7/CDDP cells by FdUMP-mediated TS inhibition. It might also be possible to assume that the duration of TS inhibition was greater in PC-7/CDDP than in PC-7 cells since an *in vivo* study suggested the importance of the duration of TS inhibition (Houghton *et al.*, 1986). However, the greater reduction in the dTTP pool in PC-7/CDDP cells has already been observed after 6 h FUra treatment. Therefore, the duration of TS inhibition may not be an important factor in the mechanism of collateral sensitivity to FUra in PC-7/CDDP cells.

Although the total TS content of PC-7/CDDP cells was 70% of that of PC-7 cells, Scanlon and Kashani-Sabet (1988) reported that a CDDP-resistant ovarian carcinoma cell line, A2780$^{CP}$, exhibited a 3-fold increase in mRNA for TS and dihydrofolate reductase and a 2.5-fold increase in the activities of both enzymes compared with the parental cells. These CDDP-resistant A2780$^{CP}$ cells showed cross-resistance to FUra, FdUrd and methotrexate (Lu *et al.*, 1988, Newman *et al.*, 1988). Teicher *et al.* (1986) reported that a CDDP-resistant head and neck squamous cell carcinoma cell line (SCC-25/CP) showed cross-resistance to methotrexate, but not to FUra. In addition, Kikuchi *et al.* (1988) reported that a CDDP-resistant cell line, KFr, derived from human serous cystadenocarcinoma of the ovary, showed no cross-resistance to FUra. Accordingly, the determinants relating to the sensitivity or resistance to FUra in CDDP-resistant cell lines might differ with the cell line.

The greater reduction in dTTP pools in PC-7/CDDP cells suggests the decrease in capacity of dTTP synthesis by a non-TS mediated process. dTMP can be synthesised from dThd rather than from dUMP in *de novo* synthesis. Accordingly, another proposed determinant of dTTP production is the availability of dThd through the salvage pathway (Nottebrook & Then, 1977; Howell *et al.*, 1978; Sobrero & Bertino, 1986). We previously showed that less exogenous $^3$H-dThd (50 nM) was incorporated into DNA of PC-7/CDDP cells than into that of PC-7 cells, without a significant difference in dThd kinase activity (Ohe *et al.*, 1990). Therefore, we examined the membrane transport of dThd, the initial process of the dThd salvage pathway. Short-term uptake of dThd was lower in PC-7/CDDP cells than in PC-7 cells. As shown in Figure 6a, it is clear that uptake of dThd was decreased in PC-7/CDDP cells. The uptake of dThd for 5 s does not mean the initial rate of dThd uptake because dThd uptake was saturated within 3 s at 25°C. However, it is possible that the difference in dThd uptake for 5 s was reflected in the difference in the membrane transport of dThd since the rate of dThd uptake was low in the second phase as shown in the time course experiment. A decreased initial uptake of dThd in the linear phase was also observed in PC-7/CDDP cells at 15°C. As shown in Figure 6b, the difference in the dThd uptake between PC-7/CDDP and PC-7 was greater in the lower exogenous dThd concentrations at 25°C. This phenomenon is consistent with the concentration response of dThd suppression of FUra-induced cytotoxicity seen in the colony-forming assay. Lower concentrations of dhd (0.1 and 1 μM) did not modify the FUra-induced cytotoxicity in PC-7/CDDP cells, and higher concentrations (10 and 30 μM) were necessary for the modification of the cytotoxicity (Table II). These data clearly demonstrate that the rate of dThd uptake at a low concentration (1 μM or less) is too low to support DNA replication in the absence of endogenous dTMP synthesis. Therefore, it is possible that FUra-induced cytotoxicity in PC-7/CDDP cells is associated with the inhibition of dTTP synthesis and that the decreased uptake of dThd is the mechanism of the collateral sensitivity to FUra in PC-7/CDDP cells.

Two dThd-membrane transport systems have been identified in mammalian cells (Belt, 1983; Vijayalakshmi & Belt, 1988; Jarvis, 1989; Plagemann & Woffendin, 1989). One is a symmetrical, non-concentrative and facilitated diffusion system with broad substrate specificity which has low affinity for dThd. The other is a sodium- and energy-dependent and concentrative transport system which has high affinity for dThd. It may be that the exogenous dThd passes through the cell membrane mainly by the high-affinity transporter in our culture condition. In addition, further analysis of dThd-membrane transport showed that the difference in the levels of dThd uptake disappeared at low temperature and an inhibitor of facilitated diffusion, DP, inhibited dThd uptake in PC-7/CDDP and PC-7 cells to a similar extent. Another inhibitor, 6-[(4-nitrobenzyl)thio]-9-β-D-ribofurasylpurine also inhibited dThd uptake by both cell line (data not shown). These data suggest that a dThd-transporter sensitive to these inhibitors exists not only in PC-7 cells but also in PC-7/CDDP cells. Therefore, we now hypothesise that the decreased uptake of dThd is related to the difference in the energy-dependent transport of dThd. However, more detailed analysis is required to elucidate the mechanisms of the decreased uptake of dThd in PC-7/CDDP cells.

Recently, there have been many studies that demonstrate a qualitative inverse relationship between drug sensitivity to CDDP and total intracellular CDDP concentration in many CDDP-resistant cell lines, exceptions to which include A2780$^{CP}$ cells (Richon *et al.*, 1987; Waund, 1987; Andrews *et al.*, 1988; Hospers *et al.*, 1988; Kraker & Moore, 1988; Kuppen *et al.*, 1988; Newman *et al.*, 1988; Bungo *et al.*, 1990; Fujiwara *et al.*, 1990). Interestingly, the accumulation of CDDP in PC-7/CDDP cells was 30% of that in PC-7 cells (unpublished data). Although CDDP is believed to enter cells by passive diffusion, an energy-dependent CDDP accumulation mechanism has been demonstrated (Andrews *et al.*, 1988). It has also been suggested that alteration in drug uptake might be one of the simplest and earliest events leading to CDDP resistance (Richon *et al.*, 1987). These findings further suggest a possibility that the decreased uptake of dThd is associated with the decreased uptake of CDDP in PC-7/CDDP cells. Although the mechanisms underlying the decreased uptake are still unclear, we speculate that common changed process(es) exist in CDDP and dThd uptake. The possibility of such factors related to uptake of CDDP and dThd warrants further study in order to clarify the mechanisms of drug resistance.

This research was supported in part by a Grant-in Aid from the Ministry of Health and Welfare for a Comprehensive 10-year Strategy for Cancer Control and from the Ministry of Education, Science, and Culture, Japan. The authors would like to thank Professor John S. Lazo (University of Pittsburgh School of Medicine) and Professor Minoru Amano (Hiroshima University) for critical reading of the manuscript. We would also like to thank Dr Youcef M. Rustum (Roswell Park Memorial Institute) for his valuable advice and discussion.

### References

ANDREWS, P.A., VELURY, S., MANN, S.C. & HOWELL, S.B. (1988). cis-Diamminedichloroplatinum (II) accumulation in sensitive and resistant human ovarian carcinoma cells. *Cancer Res.*, **48**, 68–73

BELT, J.A. (1983). Heterogeneity of nucleoside transport in mammalian cells: two types of transport activity in L1210 and other cultured neoplastic cells. *Mol. Pharmacol.*, **24**, 479–484.

BUNGO, M., FUJIWARA, Y., KASAHARA, K., NAGAGAWA, K., OHE, Y., SASAKI, Y., IRINO, S. & SAIJO, N. (1990). Decreased accumulation as a mechanism of resistance to cis-diamminedichloroplatinum (II) in human non-small cell lung cancer cell lines: relation to DNA damage and repair. *Cancer Res.*, **50**, 2549–2553.

CHABNER, B.A. (1981). Pyrimidine antagonists. In *Pharmacologic Basis of Cancer Treatment* Chabner, B.A. (ed.) pp. 183–212. The W.B. Saunders Co.: Philadelphia.

EVANS, R.M., LASKIN, J.D. & HAKALA, M.T. (1980). Assessment of growth-limiting events caused by 5-fluorouracil in mouse cells and in human cells. *Cancer Res.*, **40**, 4113–4112.

FRAM, R.J., WODA, B.A., WILSON, J.M. & ROBICHAUD, N. (1990). Characterization of acquired resistance to cis-diamminedichloroplatinum (II) in BE human colon carcinoma cells. *Cancer Res.*, **50**, 72–77.

FUJIWARA, Y., SUGIMOTO, Y., KASAHARA, K., BUNGO, M., YAMAKIDO, M., TEW, K.D. & SAIJO, N. (1990). Determinants of drug response in a cisplatin-resistant human lung cancer cell line. *Jpn. J. Cancer Res.*, **81**, 527–535.

HEIDELBERGER, C., DANENBERG, P.V. & MORAN, R.G. (1983). Fluorinated pyrimidines and their nucleosides. In *Advances in Enzymology and Related Areas in Molecular Biology* Meister, A. (ed.) pp. 57–119. John Wiley & Sons: New York.

HONG, W.S., SAIJO, N., SASAKI, Y., MINATO, K., NAKANO, H., NAKAGAWA, K., FUJIWARA, Y., NOMURA, K. & TWENTYMAN, P.R. (1988). Establishment and characterization of cisplatin-resistant sublines of human lung cancer cell lines. *Int. J. Cancer*, **41**, 462–467.

HOSPERS, G.A.P., MULDER, N.H., DE JONG, B. DE LEY, L., UGES, D.R.A., FICHTINGER-SCHEPMAN, A.M.J., SCHEPER, R.J. & DE VRIES, E.G.E. (1988). Characterization of a human small cell lung carcinoma cell line with acquired resistance to cis-diamminedichloroplatinum (II) *in vitro*. *Cancer Res.*, **48**, 6803–6807.

HOUGHTON, J.A., WEISS, K.D., WILLIAMS, L.G., TORRANCE, P.M. & HOUGHTON, P.J. (1986). Relationship between 5-fluoro-2′-deoxyuridylate, 2′-deoxyuridylate, and thymidylate synthase activity subsequent to 5-fluorouracil administration, in xenografts of human colon adenocarcinomas. *Biochem. Pharmacol.*, **35**, 1351–1358.

HOWELL, S.B., ENSMINGER, W.D., KRISHAN, A. & FREI, A. (1978). Thymidine rescue of high-dose methotrexate in humans. *Cancer Res.*, **38**, 325–330.

JACKMAN, A.L., TAYLOR, G.A., CALVERT, H. & HARRAP, K.R. (1984). Modulation of anti-metabolite effects: effect of thymidine on the efficacy of the quinazoline-based thymidylate synthetase inhibitor, CB3717. *Biochem. Pharmacol.*, **33**, 3269–3275.

JARVIS, S.M. (1989). Characterization of sodium-dependent nucleoside transport in rabbit intestinal brush border-membrane vesicles. *Biochim. Biophys. Acta*, **979**, 132–138.

KEYOMARSI, K. & MORAN, R.G. (1986). Folinic acid augmentation of the effects of fluoropyrimidines on murine and human leukemic cells. *Cancer Res.*, **46**, 5229–5235.

KIKUCHI, Y., IWANO, I., MIYAUCHI, M., KITA, T., OOMORI, K., KIZAWA, I., SUGUTA, M. & TENJIN, Y. (1988). The mechanism of acquired resistance to cisplatin by a human ovarian cancer cell line. *Jpn. J. Cancer Res.*, **79**, 632–635.

KOHN, K.W., ERICKSON, L.C., EWIG, R.A.G. & FRIEDMAN, C.A. (1976). Fractionation of DNA from mammalian cells by alkaline elution. *Biochemistry*, **15**, 4629–4637.

KOHN, K.W., FRIEDMAN, C.A., EWIG, R.A.G. & IQBAL, Z.M. (1974). DNA chain growth during replication of asynchronous L1210 cells. Alkaline elution of large DNA segments from cells lysed on filters. *Biochemistry*, **13**, 4134–4139.

KRAKER, A.J. & MOORE, C.W. (1988). Accumulation of cis-diamminedichloroplatinum (II) and platinum analogues by platinum-resistant murine leukemia cells *in vitro*. *Cancer Res.*, **48**, 9–13.

KUPPEN, P.J.K., SCHUITEMAKER, H., VANT'T VEER, L.J., DE BRUIJN, E.A., VAN OOSTEROM, A.T. & SCHRIER, R.I. (1988). cis-Diamminedichloroplatinum (II)-resistant sublines derived from two human ovarian tumor cell lines. *Cancer Res.*, **48**, 3355–3359.

LÖNN, U. & LÖNN, S. (1986). DNA lesions in human neoplastic cells and cytotoxicity of 5-fluoropyrimidines. *Cancer Res.*, **46**, 3866–3870.

LÖNN, U. & LÖNN, S. (1988). Increased levels of DNA lesions induced by leucovorin-5-fluoropyrimidine in human colon adenocarcinoma. *Cancer Res.*, **48**, 4153–4157.

LU, Y., HAN, J. & SCANLON, K.J. (1988). Biochemical and molecular properties of cisplatin-resistant A2780 cells grown in folinic acid. *J. Biol. Chem.*, **263**, 4891–4894.

MAJOR, P.P., EGAN, E., HERRICH, D. & KUFE, D.W. (1982). 5-Fluorouracil incorporation in DNA of human breast carcinoma cells. *Cancer Res.*, **42**, 3005–3009.

NEWMAN, E.M., LU, Y., KASHAMI-SABET, M., KESAVAN, V. & SCANLON, K.J. (1988). Mechanism of cross-resistance to methotrexate and 5-fluorouracil in an A2780 human ovarian carcinoma cell subline resistant to cisplatin. *Biochem. Pharmacol.*, **37**, 443–447.

NOTTEBROCK, H. & THEN, R. (1977). Thymidine concentrations in serum and urine of different animal species and man. *Biochem. Pharmacol.*, **26**, 2175–2179.

OGUR, M. & ROSEN, G. (1950). The nucleic acids of plant tissues. I. The extraction and estimation of desoxypentose nucleic acid and pentose nucleic acid. *Arch. Biochem. Biophys.*, **25**, 262–276.

OHE, Y., SUGIMOTO, Y. & SAIJO, J. (1990). Collateral sensitivity of cisplatin-resistant human non-small lung cancer cell lines to thymidylate synthase inhibitors. *Cancer J.*, **3**, 332–336.

OHE, Y., NAKAGAWA, K., FUJIWARA, Y., SASAKI, S., MINATO, K., BUNGO, M., NIIMI, S., HORICHI, N., FUKUDA, M. & SAIJO, N. (1989). *In vitro* evaluation of the new anticancer agents KT6149, MX-2, SM5887, menogaril, and liblomycin using cisplatin- or adriamycin-resistant human cancer cell lines. *Cancer Res.*, **49**, 4098–4102.

PAGOLOTTI, A.L. Jr, NOLAN, P.A. & SANTI, D.V. (1981). Methods for the complete analysis of 5-fluorouracil metabolites in cell extracts. *Anal. Biochem.*, **117**, 178–186.

PARK, J.-G., COLLINS, J.M., GAZDAR, A.F., ALLEGRA, C.J., STEINBERG, S.M., GREENE, R.F. & KRAMER, B.S. (1988). Enhancement of fluorinated pyrimidine-induced cytotoxicity by leucovorin in human colorectal carcinoma cell lines. *J. Natl Cancer Inst.*, **80**, 1560–1564.

PLAGEMANN, P.G.W. & WOFFENDIN, C. (1989). $Na^+$-dependent and -independent transport of uridine and its phosphorylation in mouse spleen cells. *Biochim. Biophys. Acta*, **981**, 315–325.

RICHON, V.M., SCHULTE, N. & EASTMAN, A. (1987). Multiple mechanisms of resistance to cis-diamminedichloroplatinum (II) in murine leukemia L1210 cells. *Cancer Res.*, **47**, 2056–2061.

SAWYER, R.C., STOLFI, R.L., MARTIN, D.S. & SPIEGELMAN, S. (1984). Incorporation of 5-fluorouracil into murine bone marrow DNA *in vivo*. *Cancer Res.*, **44**, 1847–1851.

SCANLON, K.J. & KASHANI-SABET, M. (1988). Elevated expression of thymidylate synthase cycle genes in cisplatin-resistant human ovarian carcinoma A2780 cells. *Proc. Natl Acad. Sci. USA*, **85**, 650–653.

SCHAER, J.-C., MAURER, U. & SCHINDLER, R. (1978). Determination of thymidine in serum used for cell culture media. *Exp. Cell. Biol.*, **46**, 1–10.

SOBRERO, A.F. & BERTINO, J.R. (1986). Endogenous thymidine and hypoxanthine are sources of error in evaluating methotrexate cytotoxicity by clonogenic assays using undialyzed fetal calf bovine serum. *Int. J. Cell Cloning*, **4**, 51–62.

TANAKA, K., YOSHIOKA, A., TANAKA, S. & WATAYA, Y. (1984). An improved method for the quantitative determination of deoxyribonucleoside triphosphates in cell extracts. *Anal. Biochem.*, **139**, 35–41.

TATSUMI, K., FUKUSHIMA, M., SHIRASAKA, T. & FUJII, S. (1987). Inhibitory effects of pyrimidine, barbituric acid and pyridine derivatives on 5-fluorouracil degradation in rat liver extracts. *Jpn. J. Cancer Res. (Gann)*, **78**, 748–755.

TEICHER, B.A., CUCCHI, C.A., LEE, J.B., FLATOW, J.L., ROSOWSKY, A. & FREI, E. (1986). Alkylating agents: *In vitro* studies of cross-resistance patterns in human cell lines. *Cancer Res.*, **46**, 4379–4383.

VIJAYALAKSHMI, D. & BELT, J.A. (1988). Sodium-dependent nucleoside transport in mouse intestinal epithelial cells. *J. Biol. Chem.*, **263**, 19419–19423.

WAUD, W.R. (1987). Differential uptake of cis-diamminedichloroplatinum (II) by sensitive and resistant murine L1210 leukemia cells. *Cancer Res.*, **47**, 6549–6555.

YOSHIOKA, A., TANAKA, S., HIRAOKA, O., KOYAMA, Y., HIROTA, Y., AYUSAWA, D., SENO, T., GARRETT, C. & WATAYA, Y. (1987). Deoxyribonucleoside triphosphate imbalance; 5-fluorodeoxyuridine-induced DNA double strand breaks in mouse FM3A cells and the mechanism of cell death. *J. Biol. Chem.*, **262**, 8235–8241.